# Releases of Fire-Derived Contaminants from Polymer Pipes Made of Polyvinyl Chloride

**DOI:** 10.3390/toxics7040057

**Published:** 2019-11-11

**Authors:** Ngee Sing Chong, Saidi Abdulramoni, Dwight Patterson, Heather Brown

**Affiliations:** 1 Department of Chemistry, Middle Tennessee State University, Murfreesboro, TN 37132, USA; Dwight.Patterson@mtsu.edu; 2 Dow Chemical, Indianapolis, IN 46268, USA; saa6f@mtmail.mtsu.edu; 3 School of Concrete and Industry Management, Middle Tennessee State University, Murfreesboro, TN 37132, USA; Heather.Brown@mtsu.edu

**Keywords:** polymer-derived contaminants, emissions analysis of PVC fires, post-fire PVC leachate characterization, GC-MS and infrared analysis of PVC-related pollutants

## Abstract

In order to assess the human exposure risks from the release of contaminants from water pipes made of polyvinyl chloride (PVC), experiments were carried out by subjecting the PVC pipe material to burning and leaching conditions followed by analysis of the emission and leachate samples. The emissions of burning pipes were analyzed by both infrared spectrometry and gas chromatography-mass spectrometry (GC-MS). The emission test results indicate the presence of chlorinated components including chlorine dioxide, methyl chloride, methylene chloride, allyl chloride, vinyl chloride, ethyl chloride, 1-chlorobutane, tetrachloroethylene, chlorobenzene, and hydrogen chloride were detected in the emissions of burning PVC pipes. Furthermore, the concentrations of benzene, 1,3-butadiene, methyl methacrylate, carbon monoxide, acrolein, and formaldehyde were found at levels capable of affecting human health adversely. The analysis of PVC pipe leachates using GC-MS shows that there are 40–60 tentatively identified compounds, mostly long-chain hydrocarbons such as tetradecane, hexadecane, octadecane, and docosane, were released when the burned PVC materials were soaked in deionized water for one week. Quantitative analysis shows that 2-butoxyethanol, 2-ethyl-1-hexanol, and diethyl phthalate were found in the burned PVC polymer at the average levels of 2.7, 14.0, and 3.1 micrograms per gram (μg/g) of pipe material. This study has significant implications for understanding the benzene contamination of drinking water in the aftermath of wildfires that burned polymer pipes in California.

## 1. Introduction

Plastics are non-biodegradable organic polymers of high molecular mass [1]. They are widely used in household and municipal applications such as water and sewer pipes, and electrical and vehicle equipment such as cables and wire networks [2]. High-density polyethylene (HDPE), polypropylene (PP), and polyvinylchloride (PVC) are the most common type of plastics used commercially because of their relatively lower cost and higher ignition temperature [3]. While, PVC is inherently more fire resistant because of the presence of chlorine (a flame retardant), HDPE and PP do not have inherent fire-retardant properties [3]. However, considering the extremely high combustion temperature of wildfires, the use of all three types of plastic pipes in locations or buildings prone to fire risks will pose additional serious impacts on environment, public health and infrastructure.

The 2018 wildfire in Paradise, California not only caused the loss of many lives, it also resulted in widespread contamination of the drinking water supply with carcinogenic benzene. The water contamination can be attributed to the formation of benzene during wildfire-related combustion of biomass but the contribution of benzene from burning polymer products used in municipal water pipes and residential buildings is another likely source that is investigated in this study. The average benzene concentration found in the drinking water of Paradise is 31 parts per billion (ppb), which exceeds both the California maximum allowable level of 1 ppb benzene and the U.S. Environmental Protection Agency’s maximum contaminant level standard of 5 ppb for benzene. The cost of water pipe repair and replacement is estimated at 300 million dollars and residents were advised not to drink, cook, and bathe in the water from the tap [4]. Another wildfire happened in Santa Rosa, California in October 2017. The fire also damaged the entire underground water-delivery system in Fountain Grove, resulting in benzene contamination of drinking water. Despite the widely known fact that different types of polymer pipes are combustible, PVC pipe is still being considered for the replacement of burned pipes because it has a low odor threshold for water and greater flame-retardant properties than HDPE and PP [5,6]. Therefore, it is important to investigate the release of benzene and other contaminants into drinking water as a result of burned PVC pipes.

Persistent high temperature due to structural fires of buildings or wildfires of burning biomass results in incomplete combustion of underground plastic pipes [7]. These combustion by-products including carbon monoxide, volatile compounds, soot particles, and solid residual ash are drawn into the pipes as a result of the vacuum created by the intensive use of water for suppressing wildfires. Therefore, the melted or burned underground pipes could contaminate the surrounding soil and the water-distribution system with toxic substances during and after wildfire episodes. These pollutants include volatile organic compounds (VOCs), particulate matter, heavy metals, polycyclic aromatic hydrocarbons (PAHs), polychlorinated dibenzofurans, and dioxins [1,2,7]. Many of these compounds are either carcinogens such as benzene, benzo(a)pyrene, and lead or endocrine disruptors with irreversible health effects [7]. The goals of this study are mainly aimed at (i) the characterization of contaminants emitted into air when PVC water pipe is subjected to burning, (ii) the identification and quantitative determination of contaminants leached into water from PVC pipes that were damaged after burning, and (iii) the application of analytical methods based on infrared spectrometry and GC-MS analysis for characterizing emission and leachate samples of PVC pipes.

## 2. Materials and Methods

### 2.1. Infrared Spectrocopy and Thermogravimetry Analysis of PVC Pipe

A Thermo iS-50 Fourier transform infrared (FTIR) spectrometer with a deuterated triglycine sulfate detector was used to confirm the composition of the PVC pipe by using an attenuated total reflectance accessory (Thermo Fisher Scientific Inc., Waltham, MA, USA). The FTIR spectrometer resolution of 4 cm^−1^ was used with 32 co-added scans. The thermal degradation profile of the PVC pipe sample was analyzed by a Q500 Model thermogravimetric analyzer (TA Instruments, New Castle, DE, USA) was used to characterize the rate of mass loss as a function of temperature from 20 °C to 1000 °C.

### 2.2. Analysis of Leachates from Burned PVC Pipes by GC-MS

Pipe samples of about 2 inch by 4 inch section was cut from PVC sewer pipes with ASTM F794 specifications. They were weighed to the accuracy of ±0.1 mg on an analytical balance in order to allow the calculation of the concentrations of water contaminants in parts-per-million (ppm) units or milligram (mg) of analyte per kilogram (kg) of PVC pipe in the leaching test. About 10–15 g of each type of pipe materials were soaked in the 300 mL of high-purity deionized water for 1 week with sonication times of 30 min twice daily. A Shimadzu QP2010 GC-MS equipped with an autosampler was used to analyze the leachates from the unburned and burned pipes after performing solid phase extraction of the contaminants with both methylene chloride and methanol. Chemical standards used for the quantitation of selected contaminants leached from the pipes include a homologous series of C10, C12, C14, C16, C18, C20, C22, and C24 n-alkanes as well as 2-butoxyethanol, and 2-ethyl-1-hexanol purchased from Sigma Aldrich (St. Louis, MO, USA), 7,9-di-tert-butyl-1-oxaspiro(4,5)deca-6,9-diene-2,8-dione (Toronto Chemical, Toronto, ON, Canada), and the EPA 8270 semi-volatile standard (AccuStandard, New Haven, CT, USA).

The Shimadzu QP2010S GC-MS was used in a splitless injection mode to analyze 1.0 μL SPE eluate from the solid phase extraction of leachate from the PVC pipe after being soaked in deionized distilled water. The GC column was a ZB5-HT (Phenomenex, Bellefonte, PA, USA) polyimide-coated fused silica column 5% phenyl/95% dimethylpolysiloxane 30 m × 0.25 mm i.d., film thickness 0.25 μm. The GC injector temperature was kept at 250 °C and splitless injection method was used at a column flow of 1.22 mL/min and a linear flow velocity of 40.1 cm/s followed by three stages of temperature ramp-up. First, the temperature was increased from 50 °C to 150 °C at 5 °C/min and held for 1 min, then to 220 °C at 15 °C/min and held for 3 min, and finally to 320 °C at 25 °C/min and held for 5 min. The mass spectrometer was operated in the scan mode with its GC interface temperature set at 320 °C and the MS ion source temperature kept at 200 °C. Mass spectra were acquired in the mass range of 29–450 amu.

### 2.3. Analysis of Emissions from Burning PVC Pipes by GC-MS

A PVC sewer pipe with ASTM F794 specifications was cut into pieces weighing 15–20 g that were used for both burn emissions and water leaching tests. A propane torch was used to start the burning of the PVC pipe material and, when the fire on the pipe became self-sustaining, the emitted gases were collected into a 3-L Tedlar^®^ bag (SKC Inc., Eighty Four, PA, USA) via the Vac-U-Chamber (SKC Inc., Eighty Four, PA, USA).

The PVC pipe emissions were analyzed using an Agilent 6890 gas chromatograph coupled to an Agilent 5973 quadrupole mass spectrometer (GC-MS) (Agilent Technologies, Santa Clara, CA, USA). A 16-position Nutech autosampler for automated sequential mode analysis was used to introduce PVC pipe emission samples of 20.0 mL into the Agilent GC-MS via the Nutech 8900DS preconcentrator (GD Environmental Supplies, Inc., Richardson, TX, USA). The preconcentrator has three cryogenic traps, the glass bead trap, Tenax TA trap and the cryofocuser, for selective enrichment of the VOCs in the emission samples by controlling the temperature and desorption time settings. An accessory called GC Chaser™ from Zip Scientific (Fast GC Technology, Goffstown, NH, USA) was used to improve the GC oven ventilation during cool-down cycles. The preconcentrator was set to preheat to 10 °C, whereas the cooling temperature was set at −150 °C, the desorption temperature at 20 °C with preheat time 2 s, and desorption flow at 15 mL/min with desorption time 2 min. The GC separation was carried out using the Rxi-1ms capillary column (Restek, Bellefonte, PA, USA) with the stationary phase of 100% polydimethylsiloxane and column parameters of 60 m × 0.32 mm i.d. and a film thickness of 1.00 μm. Initially, the GC oven was set at 30 °C for 3 min before undergoing three sequential temperature ramps without any hold, from 30 °C to 100 °C at 5 °C/min, from 100 °C to 150 °C at 12 °C/min, and from 150 °C to 200 °C at 15 °C/min. The final temperature of 200 °C was held for 4 min for a total GC run time of 28.50 min. The GC inlet temperature was maintained at 320 °C and the helium carrier gas flowrate was set at 28 cm/s. Mass spectrometer was operated in the simultaneous full scan and selected ion monitoring (SIM) modes. The electron impact ionization was operated at 70 eV electron energy. The interface temperature was set at 320 °C and mass spectra were recorded in the m/z range of 35–350 amu.

The analytical procedures for the determination of VOCs were similar to those in the USEPA TO-15 method described in the Compendium of Air Toxics. The detection limits of 0.1–3 parts per billion by volume (ppbv) were achieved for the 72 target compounds of the TO-15 method and also all the other compounds found in the emissions of burning polymer pipes or headspace vapor of burned polymer materials. Evacuated sampling bottles were used to hold the burned pieces before being connected to a 16-position autosampler for the pre-concentrator. The identification of VOCs was based on the Agilent ChemStation software with the 2017 mass spectral database developed by National Institute of Standards and Technology (NIST, Gaithersburg, MD, USA). Identification of compounds were performed by both mass spectral search with match indices of greater than 70 and retention index window of ±50 units. Quantitation was based on linear regression analysis of the analytical responses of standards at 4–7 levels. For both tetradecane and hexadecane, a polynomial function was used for quantitative calibration.

### 2.4. Analysis of Emissions from Burning PVC Pipes by Infrared Spectrometry

For the pipe emission analysis of very volatile compounds, typically gases with molecular mass of less than 50 amu, a Varian 7000 Fourier transform infrared (FTIR) spectrometer with a mercury–cadmium–telluride detector cooled by liquid nitrogen was used. A Tornado 10-meter gas cell (Specac, Orpington, UK) with gold-coated mirrors was used to achieve an optical pathlength of 10 m via multiple reflections of the infrared beam through the gas samples of pipe emission. The infrared spectra were acquired with the Happ-Genzel apodization function and the spectrometer resolution of 0.5 cm^−1^ for 32 co-added scans. Quantitative analysis was carried by the univariate approach of comparing the sample spectra with the standard reference spectra of a commercially available infrared spectral database (Infrared Analysis, Anaheim, CA, USA). The average concentration values determined at 3-5 different wavenumber regions of FTIR spectra. The determination was based on peak areas when spectral overlap was minimal but relied on peak height when spectral overlap was suspected. Compounds analyzed by the FTIR method include ethylene, acetylene, formaldehyde, methane, ethane, carbon monoxide, chlorine dioxide, and hydrogen chloride.

## 3. Results

The results of this study are aimed at characterizing the profile of contaminants that can be released in the combustion of PVC sewer pipes with the ASTM F794 specifications. This material, analyzed by both FTIR and TGA, shares many chemical characteristics with common household or municipal PVC pipes that may be burned in structural or wildland fires. The toxicants that are produced in wildfire have adverse impact on human health via two exposure pathways. The ingestion pathway can be attributed to the leaching of the contaminants from the burned and thermally degraded PVC pipes. The contaminants can remain in the water system for months and may pose health risks to residents who use the water for drinking, cooking, and bathing. The direct inhalation of toxicants from the burning of PVC pipes is associated with adverse respiratory effects of asthma, bronchitis, and dizziness through short term exposure to emissions of the wildfires. These undesirable effects of inhalation become less severe in the ensuing days in the aftermath of the wildfire because the air pollutants are rapidly diluted in the atmosphere. These two exposure pathways are discussed below.

### 3.1. Thermogravimetric Characterization of PVC Pipes

The pipe material was confirmed to be predominantly PVC by comparing the infrared spectra of the pipe samples with spectra in commercial infrared spectral libraries. The match index of the 91.72 out of the maximum of 100 was achieved for a PVC pipe reference spectrum in the Common Materials Library but the match index for high purity PVC infrared spectrum in the Hummel Sadtler Library is only 75.41 out of 100. This indicates that there are significant amounts of fillers or additives used in the PVC pipe formulation. The TGA characterization of PVC pipe samples shows that there are four distinct steps of mass loss as the PVC pipe sample is heated from 20 °C in nitrogen atmosphere, switching to air at 50 °C, and continued heating in air to 1000 °C. The first step of PVC thermal degradation occurred at 210–245 °C and was associated with 39.98% (*w/w*) of mass loss due to the dehydrochlorination reaction. The second step of 18.33% mass loss (245–300 °C), almost seen as a continuation of the first step, is likely due to dehydrochlorination at the cross-linked and sterically hindered portion of the polymer because the loss of HCl from the (CH_3_Cl)_n_ represents 58.4% by weight, which is almost equal to the sum of weight loss percentages of the first two steps of thermal degradation. The mass loss of 18.31% occurred consecutively at up to 450 °C with the last step of 19.25% mass loss ending at about 700 °C. This last two steps of loss is related to evolution of long-chain hydrocarbons, siloxanes and CO_2_. The residue left at 1000 °C was determined by FTIR to be a mixture of calcium carbonate and silicates that had been used as a filler for PVC pipe production.

### 3.2. Leachate Analysis for Polymer Pipes

The GC-MS data show that close to 100 compounds are leached from the PVC polymer pipes into the water during the leaching test. The number of compounds in each class of organic compounds follows the trend of alkanes≈alkenes > aromatics > oxygenated additives > cycloalkanes and cycloalkenes > halogenated compounds. The compounds quantified in the leachate of the polymer pipes are shown in Table 1 below. The significance of the data in Table 1 is to compare not only the chemical profiles of burned and unburned PVC pipe leachate but also to show how their leachate composition is different from the composition of compounds emitted into the gas phase during the burning of PVC pipes. In general, the leachate constituents are polar semi-volatile compounds that include polymer additives whereas the pipe emissions due to burning consist of volatile compounds that are primarily alkenes, aromatics, and chlorine-containing compounds like hydrogen chloride, chlorine dioxide, 1-chlorobutane, vinyl chloride, ethylene dichloride, and chlorobenzene.

The leachate compounds of the greatest toxicological concern are the phthalate esters, which include diethyl phthalate found at the level of 2.4 μg/g and diisooctyl phthalate at 3.0 μg/g for the unburned PVC pipe. Both phthalate esters are endocrine disruptors that interfere with the body’s endocrine system and produce adverse developmental, reproductive, neurological, and immune effects in both humans and wildlife. The release of endocrine disruptors from polymer pipes used for drinking water distribution is potentially harmful to human health because of the daily low-dose exposure. Adverse health effects observed include lowered fertility rate, an increased incidence of endometriosis and some forms of cancers [8,9,10,11]. Another chemical that is potentially harmful but has received much less attention is 7,9-di-tert-butyl-1-oxaspiro(4,5)deca-6,9-diene-2,8-dione. This compound has been investigated as a contaminant in maple syrup [12], ophthalmic drug product [13], and water of Prut River on the Romanian and Moldavian border [14]. In all three cases, the sources of this contaminant were respectively linked to plastic conduits for the flow of maple syrup from the trees to the collector vessel, the plastic container for the ophthalmic solutions, and the plastic waste discarded into the Prut River. There is no published or online toxicological information related to 7,9-di-tert-butyl-1-oxaspiro(4,5)deca-6,9-diene-2,8-dione but it was reported as a transformation product of Irganox 1010, which is commonly used as an antioxidant additive in polymer pipes used in water distribution [15].

2-Butoxyethanol was found in the leachates of PVC polymer at the concentrations of 2.7 μg/g and 2.8 μg/g in burned and unburned pipes. It can enter our body through inhalation or absorption through our stomach and intestines when consuming food or water that is contaminated. The harmful effect most often reported in animals exposed to 2-butoxyethanol is destruction of red blood cells that results in the release of hemoglobin, which is known as hemolysis. Effects related to hemolysis include increased hemoglobin levels in the urine, blood in the urine, and the build-up of hemoglobin and destroyed red blood cells in organs such as the kidney, spleen, and liver [16]. 2-Ethylhexanol has been found in exhaled breath and adipose tissue due to its widespread use in 2-ethylhexyl nitrate as a cetane improver and in diethylhexyl phthalate as a plasticizer additive. It was found in the PVC leachate samples at 11.6 μg/g and 14.0 μg/g for the unburned and burned pipes. It is a peroxisome proliferator and a metabolite of diethylhexyl phthalate, which causes peroxisome proliferation in animal studies and liver tumors. Liver tumors were produced with 2-ethylhexanol in female mice that had been given more than the maximum tolerated dose of 750 mg/kg body weight [17]. Straight-chain alkanes including tetradecane, hexadecane, octadecane, and docosane were reported in Table 1 below. Their levels range from 1.1 μg/g for octadecane to 27.8 μg/g for tetradecane for the burned PVC material. These alkanes are generally not too harmful to human health and have not been studied much.

### 3.3. Emission Analysis of Burning Polymer Pipes

Although the testing shows the detection of >100 contaminants under different treatment scenarios, only selected contaminants with known toxicological effects and those that are regulated by Occupational Safety and Health Administration (OSHA) and the U. S. Environmental Protection Agency (USEPA) are tabulated in Table 2 below. The Permissible Exposure Limits (PEL) in Table 2 are OSHA standards intended to protect the health of workers in industrial settings. In the context of health threats posed by wildfires, PEL values are of great significance in safeguarding the health of firefighters and other first responders in wildfire emergency management. Both the cancer target risk and non-cancer hazard index are health-based screening levels for risk assessment of toxicant exposure for the general population or residents impacted by wildfires. The cancer target risk value of 1 × 10^−6^ refers to the air toxicant concentration that would result in the excess risk of one out of one million equally exposed people contracting cancer if exposed continuously (24 h per day) to the specific concentration over 70 years. The non-cancer hazard index is the ratio of exposure concentration in the air to the health-based reference concentration set by EPA. When the toxicant concentrations are significantly larger than the tabulated concentrations for non-cancer hazard index of 1, they are more likely to cause adverse non-cancer health effects over a lifetime of exposure. Table 2 shows that benzene and 1, 3-butadiene levels are above the PEL values and are at least 100 times higher than either the cancer target risk or non-cancer hazard index values. Although acetaldehyde and chloromethane were not found at higher levels than the PELs, they were higher than the USEPA screening levels based on cancer risk and non-cancer hazard index. The detected levels of carbon monoxide (CO) at the mean concentration of 33.8 ppmv (i.e., parts per million by volume) were greater than the PEL of 25 ppmv as well as the National Ambient Air Quality Standards of 9 ppmv for 8-h period and 35 ppmv for 1-h period. Breathing CO can cause headache, dizziness, vomiting, and nausea. Exposure to high CO levels may lead to the loss of consciousness or death while exposure to moderate to high CO levels over extended periods has been linked to increased risk of heart disease.

**Figure 1:**
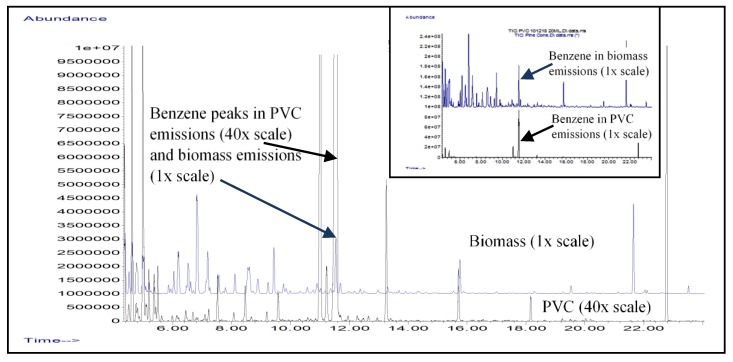
Overlaid GC-MS chromatograms of emissions from burning biomass compared to burning PVC pipes. Note that the scale for PVC emissions is expanded forty-fold to show the presence of about 85 volatile organic compounds (VOCs) detected at trace levels. The inset figure shows the same chromatograms on a common scale and the presence of about six major components in the burning PVC emissions.

Burning PVC water pipes produced emissions that contained a large number of airborne contaminants, especially those organochlorine and organosilicon compounds that are potentially harmful to human health. As shown in Figure 1 and Table 2, the PVC emissions contained extremely high concentrations of toxic compounds such as 1-butene, 1, 3-butadiene and benzene. The burning PVC pipes produced these three contaminants at concentration levels that are significantly higher than their recommended screening levels for cancer target risk and non-cancer hazard index. For PVC emissions, hydrogen chloride, chloromethane, chloroethene (vinyl chloride), 1, 3-butadiene and benzene are major contaminants of concern in the emissions of PVC burning pipes. Other PVC-derived contaminants including 1-chlorobutane, dichloroethyne, 3-chloropropyne, thirane, chlorobenzene, carbon tetrachloride, and 2,3-bis(trimethylsilyl) ether dimethylhydroquinone were also detected but not quantified due to the lack of standards. Silicon-containing compounds including 2-[(trimethylsilyl)oxy]-2-{4[(trimethylsilyl)oxy]phenyl}-ethaneamine, bis(trimethylsilyl) ether of 2,3-dimethylhydroquinone, and bis(trimethylsilyl)-3,4-dihydroxybenzyl alcohol have been tentatively identified in the burning PVC pipe emissions but these compounds have never been studied for toxicological risks. The formation of these compounds is likely related to the degradation of siloxanes and their subsequent reaction with other combustion by-products of PVC at the extremely high wildfire temperature of above 500 °C [18]. This provided the trimethylsilyl moieties for substituting onto the phenyl ring of benzene and other alkyl aromatics. Figure 1 also provides side-by-side comparison of GC-MS chromatograms for the emissions of burning PVC and burning cellulosic biomass of leaves, twigs, and pine cones. In PVC emissions data, benzene is the most abundant component accounting for 54% of the GC-MS signals measured among the 88 VOCs detected. In comparison, the cellulosic emissions show benzene only accounting for 4.8% of very complex emission profile with about 184 VOCs detected. The comparison of fire-derived contaminant profiles from the combustion of PVC pipe and biomass would be useful in determining their relative degrees of contribution in drinking water contamination. The presence of HCl, ClO_2_, chlorinated hydrocarbons, and siloxane compounds are characteristic of PVC pipe emissions whereas the presence of terpenes their degradation compounds including limonene, 3-carene, α-pinene, isoprene, 1, 3-butadiene, 1, 3-pentadiene acrolein, methacrolein, butanedione, and 1,3-buten-1-ol are indicative of biomass emissions from wildfires.

Table 2 is provided to show the concentrations of air toxicants generated from the combustion of PVC pipe and their potential for adverse human health effects by tabulating the cancer and non-cancer risk levels from the USEPA database. Table 2 along with Figure 1 also provide clues as to the source of drinking water contaminants found in the aftermath of the wildfires in California. According to a technical memorandum issued by the City of Santa Rosa, the post-wildfire water samples were found to contain aromatic hydrocarbons such as benzene, toluene, ethylbenzene, and the xylenes, polynuclear aromatic hydrocarbons, chlorinated compounds, ketones, furans, and thiophenes [19]. With the exception of polynuclear aromatic hydrocarbons that were not analyzed in the current study, the chemical profiles of emissions from burning biomass and PVC pipes are consistent with the description of water contaminants. The leachate samples have minimal or non-detectable levels of benzene which are significantly lower than those of the contaminants reported in Table 1. This suggests that benzene is not present in the new unburned PVC pipe and the benzene emitted from burning pipes do not have much affinity to permeate within the burned PVC matrix. Both the burning biomass and burning PVC studies support the finding that the wildfire caused the thermal degradation of plastic pipes and the subsequent entry of soot and other contaminants into drinking water pipes as a result of the loss of water pressure and subsequent back-siphoning of the contaminants into water service lines.

The toxicological evaluation of the estrogenic, androgenic, and progestogenic potential of octamethylcyclotetrasiloxane has shown that it can bind to the estrogenic receptor (ERα) and activate the reporter gene at 10 mM. In both the RUA and Hershberger assays conducted for inhalation of octamethylcyclotetrasiloxane for 16 h/day, a small but significant increase in both wet and blotted uterine weight as well as increases in both luminal and glandular epithelial cell height were observed in both Sprague Dawley and Fischer 344 rats [20]. Serum estradiol levels decreased in a dose-dependent manner after being exposed to 100 mg/kg to 1000 mg/kg concentrations of octamethylcyclotetrasiloxane. Uterine peroxidase activity, a marker for estrogenic activity, was also significantly increased in mice exposed to octamethylcyclotetrasiloxane but not in mice exposed to the other siloxanes [21].

It is important to note that in Table 2, the observed airborne contaminant levels of benzene (10.5 ppmv) and 1, 3-butadiene (2.4 ppmv) found in burning PVC pipe emissions exceed the OSHA regulatory standards for occupational safety. These OSHA standards are intended to prevent harmful inhalation exposure of workers such as firefighters trying to suppress wildfires, emergency response personnel in charge of evacuating residents, and hazardous waste operators removing the burned PVC pipes. However, both of these compounds are above the screening levels of Cancer Target Risk (CTR) and Non-Cancer Hazard Index (NCHI) reported in the USEPA Integrated Risk Information System (IRIS) database. For the burning PVC pipe emissions, chloroethene or vinyl chloride and ethylbenzene are also above the CTR screening level. The PVC emissions also include a very high level of 3007 ppbv of chloromethane in comparison to its NCHI screening level of 45.5 ppbv. Other compounds such as 1-chlorobutane, 1-butene, and 2, 3-dimethylhydroquinone, bis(trimethylsilyl) ether were also found at very high levels but were not quantified since there are no toxicological information or regulatory standards.

The FTIR method was used for the analysis of very volatile compounds in the PVC pipe emissions that are not amenable to GC-MS analysis. The sample data for the PVC pipe are shown in Table 3 below. The concentration of CO is at 33.8 ppmv, which exceeds both the National Ambient Air Quality Standards (NAAQS) of CO at 9 ppmv and 35 ppmv for 8-hours and 1-hours averaging periods, respectively. Although the relatively high levels of ethylene (3.1 ppmv) and acetylene (2.0 ppmv) are not particularly of great concern in terms of health effects, they play a key role in the formation of harmful and carcinogenic compounds like benzene, formaldehyde, acetaldehyde, as well as 1, 3-butadiene. Hydrogen chloride, a sensory and pulmonary irritant, was determined to be present at 2.3 ± 0.1 ppmv in the emissions of burning PVC pipe. The dehydrochlorination reaction of burning PVC pipe at 200–300 °C is responsible for the release of hydrogen chloride. Siloxane compounds identified by GC-MS as hexamethylcyclotrisiloxane, octamethylcyclotetrasiloxane, and decamethylcyclopentasiloxane found in PVC pipe emission, was quantified as 0.462 ± 0.051 ppmv of octamethylcyclotetrasiloxane by FTIR analysis. Among the compounds measured by FTIR, formaldehyde (0.049 ppmv) and hydrogen chloride (2.3 ppmv) have great likelihood of contaminating water supply because of their high solubility values as shown in Table 3. However, both were not measured in the leaching study because both the solid phase extraction cartridge and GC-MS analysis based on the relatively non-polar stationary phase of the column are not suitable for hydrogen chloride analysis. Furthermore, the lower limit of GC-MS mass scan starting from 35 amu is not applicable for analyzing formaldehyde with a molar mass of 30 amu. Since formaldehyde is classified as Group 1 or carcinogenic to human by International Agency for Research on Cancer, future work for modifying the analytical method to detect formaldehyde in water is necessary. Depending on the actual conditions of burning, chlorine dioxide has also been detected in the emissions at a high concentration. The inhalation of chlorine dioxide can be potentially harmful because it is a powerful oxidant and may disrupt physiological processes in humans once it is absorbed into the blood stream via the alveolar tissue in the lungs.

## 4. Discussion

A previous study of the leaching or migration of compounds from polymer pipes into water shows that PVC imparts the lowest odor threshold and yields the least number of VOCs compared to HDPE and cross-linked polyethylene pipes (PEX) [22]. Although the popularity of PVC pipes can be attributed to these desirable characteristics, it may not be the best choice for water distribution in view of the recent wildfire episodes that could cause widespread contamination of drinking water quality in affected communities. The results shown in this study for the burning PVC pipes cast doubts on their ability to withstand the thermal degradation of wildfires that would cause the release of close to 100 compounds. These include four known IARC Group 1 human carcinogens including vinyl chloride, formaldehyde, 1,3-butadiene, and benzene [23]. The emissions testing also show three Group 2A probable carcinogens including methylene chloride, tetrachloroethylene, and styrene and four Group 2B possible carcinogens including chloroform, carbon tetrachloride, acetaldehyde, and ethylbenzene. Trihalomethanes such as chloroform have been linked to increased risk for developing bladder cancer [24]. While it is debatable as to whether PVC should be the pipe of choice for water distribution in regions prone to wildfires, this study brings up the important question as to whether there is an alternate pipe material that is a more fire retardant and hence poses the least health threat of fire-derived pollutants. Cement-based materials used to convey drinking water include reinforced concrete pipes, cement mortar linings and asbestos–cement (AC) pipes. AC pressure pipes gained popularity as a drinking water pipe system due to many comparable benefits to PVC—lightweight, strong and low hydraulic friction. In addition, AC pressure pipes are heat and fire resistant as well as corrosion free [25]. Due to the use of asbestos fibers, regulations have limited replacement of AC pipes due to asbestos fiber hazards to workers and modern fiber replacements are available. Cement based pipes do not have the toxicity concerns that its counterparts have and is noted in a 2002 EPA Study as having only 1% of the US incidents concerning permeation and leaching of toxic substances [26].

This research study has confirmed that benzene is the most important contaminant to be reckoned with in terms of its toxicity via inhalation or ingestion and its abundance among all the contaminants. The GC-MS results show that the benzene signal intensity accounts for 47.6% of all detected compounds in PVC pipe emissions. Another study by Huggett and Levin at the former National Bureau of Standards (NBS) has also demonstrated the presence of benzene and other chlorinated hydrocarbons [27]. However, the NBS study has no quantitative data for analyte concentrations and the number of detected compounds is less than the number reported for the current study, presumably due to the greater analytical sensitivity of the cryogenic Tenax TA sorbent trap method used. A more recent publication describing the use of a canister-based GC-MS method has detected benzene, toluene, styrene, propane, 2-methylfuran, decene, and ethylbenzene during a fire at a plastic waste recycling facility [28]. These two studies corroborate our results that benzene is a major component of the thermal degradation of plastics, especially PVC.

Although it is reasonable to use benzene concentrations in contaminated water supplies as an indication of the effectiveness of remediation efforts, further research should be aimed at the application of highly sensitive analytical methods that are capable of measuring highly toxic compounds with large Henry’s Law constants that facilitate their partition into drinking water or yield increased solubilities. Dioxins, though not very soluble in water, are highly toxic and have propensity for partitioning onto inhalable particulate matter that could enter the human food chain and bioaccumulate in adipose tissues. The maximum contaminant level (MCL) promulgated by EPA is 30 parts per quadrillion (ppq), which is about 167,000 times lower than the MCL of benzene at 5 ppb. To the best of the authors’ knowledge, there has not been any published studies on the analysis of dioxins in water samples collected in the aftermath of wildfires in Paradise or Santa Rosa in California. There is a high probability of dioxins being produced from the combustion of wildfires at the wildland-urban interface, where PVC is common in household products, building materials, and polymer pipes for distributing drinking water, storm water, and sewage water. This is corroborated by a recent review article that describes the various ways PVC has been involved in the production highly toxic dioxins [29]. These include dioxins being produced in the municipal waste incinerator, house or structure fires, landfill fires, and backyard burning because PVC materials are ubiquitous. The widespread incidence of dioxin contamination during combustion processes is partly due to the rapid conversion of the PVC into gaseous HCl at a lower temperature of thermal treatment compared to other polymers. The observation of the onset of mass loss for the PVC pipe sample at 210–245 °C in our TGA data is consistent with the TGA data reported by Bhaskar et al. that show that the decomposition or onset of mass loss for PVC takes place at about 250 °C whereas PP and HDPE show initial decomposition at 350 °C and 460 °C, respectively [30]. The emission of HCl plays a major role in the generation of many congeners of dioxins with various numbers of chlorine substituents in polychlorinated dibenzodioxins (PCDDs) and polychlorinated dibenzofurans (PCDFs). Furthermore, the PVC pipe residue obtained towards the end of our TGA test at 1000 °C shows, via FTIR analysis, the presence of both silicate and calcium carbonate materials, which are typical of clay, limestone, or kaolin fillers used in PVC pipe manufacture [31,32].

This article is likely the first that is dedicated to the study of fire-derived contaminants in both the water leachates and combustion emissions of PVC pipes. For future study, it is important to test different types of drinking water pipes that are classified as unplasticized PVC, modified PVC, oriented PVC, fusible PVC, HDPE, and PEX produced by different manufactures [33]. The different polymeric composition along with their unique additives will influence both the emission and leachate profiles. Specifically, the evaluation of National Sanitation Foundation (NSF) Standard 61 pipe for leaching potential under both combustion and pyrolysis conditions will be necessary. Future research will be targeted at the development of more versatile analytical methods capable of analyzing a wide range of compounds with different polarities or solubilities in water at sub-ppb levels. It is not known at this point which EPA methods, other than EPA Method 524, are used for the analysis of drinking water by Californian water agencies or local water utility companies after the wildfires. If the methods used are based on the EPA 524.2 or 524.3 methods involving purge-and-trap technique with GC-MS analysis, then the range of analytes amenable for analysis will be limited by the volatilities of the compounds and their compatibilities with the types of sorbents used in the trap. Ideally, MS analysis after liquid chromatography separation (LC-MS) of the more polar and less volatile compounds should be used for the determination of semi-volatile or non-volatile compounds that may also be found in drinking water. The method used in the current study involves solid phase extraction with the octadecyl sorbent phase that can capture a wide range of compounds as attested by the presence of close to 100 GC peaks, even though only 40–60 can be tentatively identified with relatively good match indices by searching against the NIST 2017 mass spectral database. Different types of sorbent materials can be used with both solid phase extraction and solid phase microextraction to determine their suitability for achieving sub-ppb detection limits of different groups of compounds. Techniques for the analysis of airborne particulate matter produced from wildfires should be evaluated for both scanning electron microscopy with energy-dispersive X-ray microanalysis of trace elements, FTIR and Raman microscopy, as well as the MS analysis of the SPE extracts of particle size fractions of less than 1.0 micron that could be inhaled and deposited in lung alveolar tissues. These ultrafine particle fractions could also be ingested because they can be dispersed in the drinking water and pass through household water filters. The ingestion of ultrafine particles in tap water has rarely been examined despite the availability of imaging methods based on infrared and Raman microscopy, scanning electron microscopy with energy-dispersive X-ray microanalysis, and laser scattering techniques that can measure the particle density at the nanoscale. In a recent review paper, only two out of fifty-six studies are related to microplastics in tap water while the majority of the studies are focused on surface and waste waters [34]. It is highly likely that the thermal degradation of PVC pipes during wildfires may generate harmful micro or nano-sized particles that are suspended in drinking water; this would warrant further investigation to determine the extent of pipe replacement needed after the California wildfires during which the water pipes were burned.

## 5. Conclusions

This study is timely considering the recent increase in the frequency and severity of wildfires in California and other western states that had caused the burning and damage of stormwater and drinking water pipes. This has led to unprecedented problems of air and water pollution that might lead to adverse human health effects via smoke inhalation and water consumption. This project has provided data and test results that will be useful for scientists, engineers, and the public to understand the potential toxicological risks that could be caused by the damage of water pipes during wildfires. Furthermore, the leachability of polymer pipes causes the release of phthalate esters and other compounds that may contribute to the bioaccumulation of endocrine disrupters, carcinogens, or other toxicants with impact on reproductive, neurological, immunological, and developmental health. Besides providing complementary data for the biomonitoring and environmental studies of phthalate esters by agencies such as the Centers for Disease Control and Prevention as well as Environmental Protection Agency in the United States, the results of this study elucidate the profile of contaminants associated with polymer-based water pipes and may encourage pipe manufacturers to develop safer and more environmentally benign pipes. The design of fire-retardant polymer pipes or pipes made of other materials will be critical in reducing human exposure to pollutants with adverse health effects. The use of thermally stable and non-leachable polymer, ductile iron, copper, or concrete materials will be highly desirable. The choice of a suitable type of water pipes that will not pose undesirable environmental or human health consequences under all circumstances, with or without occurrence of wildfires, is of the utmost importance in public health and safety.

## Figures and Tables

**Table 1:** Concentrations of contaminants in the leachates of burned and unburned polyvinyl chloride (PVC)polymer pipes shown in the units of “ppm” or “μg analyte per gram of pipe material”. The relative standard deviation of triplicate analysis is calculated to be in the range of 20–45% for the compounds detected.

Identified Compounds	Burned PVC	PVC
**Tetradecane**	27.8	21.6
**Hexadecane**	11.6	13.6
**Octadecane**	1.1	1.2
**Docosane**	9.0	5.4
2-Butoxyethanol *	2.7	2.8
Irganox 1010 constituent **	3.3	3.8
**2-Ethyl-1-hexanol**	14.0	11.6
**Diethyl phthalate**	3.1	2.4
**Diisooctyl phthalate**	<MDL ***	3.0

* The data for 2-butoxyethanol was based on solid phase extraction of the pipe leachate using methanol whereas other data were based on methylene chloride extraction. ** ”Irganox 1010 constituent” refers to 7,9-di-tert-butyl-1-oxaspiro[4,5]deca-6,9-diene-2,8-dione. *** “<MDL” means the analyte is below minimum detection limit.

**Table 2:** Concentrations of major air pollutants in the emissions of burning PVC pipes and their comparison to Occupational Safety and Health Administration (OSHA) regulatory standards for permissible exposure levels (PEL) and USEPA health screening levels based on excess cancer risk = 1 × 10^−6^ and hazard index = 1.

GC Peak (min)	Compounds	Level (ppbv)	PEL * (ppmv)	STEL * (ppmv)	Cancer Target Risk (CTR) ** = 1 × 10^−6^ (mg/m^3^; ppbv)	Non-cancer Hazard Index (NCHI) = 1 (mg/m^3^; ppbv)
4.61	Chloromethane	3007	50	100	N.A.***	9.4 × 10^1^; 45.5
4.87	Chloroethene	25	N.A.	N.A.	1.7 × 10^−1^; 0.0665	1.0 × 10^2^; 39.1
5.00	1-Butene	4885	N.A.	N.A.	N.A.	N.A.
5.02	1,3-Butadiene	2377	1	5	9.4 × 10^−2^; 0.0425	2.1; 0.95
5.49	Ethyl Chloride	506	100	N.A.	N.A.	1.0 × 10^4^; 3789
7.10	Methylene chloride	10	200	300	N.A.	5.2 × 10^3^; 1763
7.42	Carbon disulfide	4	N.A.	N.A.	N.A.	7.3 × 10^2^; 207
11.63	Benzene	10,519	1	5	3.6 × 10^−1^ (0.113 ppbv)	3.1 × 10^1^; 9.7
18.63	Chlorobenzene	10	10	N.A.	N.A.	5.2 × 10^1^; 11.3
19.23	Ethylbenzene	11	5	30	1.1 (0.253 ppbv)	1.0 × 10^3^; 230
20.19	*o*-Xylene	11	100	150	N.A.	1.0 × 10^2^; 23.0

* PEL and STEL refer to permissible exposure limits and short-term exposure limits for protecting the safety of workers. They are regulatory standards of Occupational Safety and Health Administration (OSHA). ** The Cancer Target Risk (CTR) and Non-Cancer Hazard Index (NCHI) screening levels are obtained from the United States Environmental Protection Agency (USEPA) Integrated Risk Information System (IRIS) database; the CTR and NCHI values are reported in both “mg/m^3^” and “ppbv” units for the contaminants measured by gas chromatography-mass spectrometry (GC-MS). *** Not available.

**Table 3:** Concentrations (ppmv) of very volatile compounds in the burning PVC pipes emissions analyzed by infrared spectrometry.

	Carbon monoxide CO	Ethylene C_2_H_4_	Acetylene C_2_H_2_	Hydrogen Chloride HCl	Formaldehyde HCHO	Octamethyl-cyclotetra- siloxane C_8_H_24_O_4_Si_4_
Mean ± 1sd *	33.8 ± 2.1	3.1 ± 0.3	2.0 ± 0.1	2.3 ± 0.1	0.049 ± 0.001	0.462 ± 0.051
Solubility **	27.6 mg/L	2.9 mg/L	Very low	720 g/L	40 g/L	56.2 μg/L
Vap. P. **	>760 torr	>760 torr	>760 torr	>760 torr	>760 torr	0.934 torr

* All the FTIR data are reported as the mean of 3–5 measurements with ±1 standard deviation. ** The solubility and vapor pressure values are based on the temperatures of 20–25 °C and are obtained from Wikipedia.
